# Microfluidic-based graphene field effect transistor for femtomolar detection of chlorpyrifos

**DOI:** 10.1038/s41598-018-36746-w

**Published:** 2019-01-22

**Authors:** Saurav Islam, Shruti Shukla, Vivek K. Bajpai, Young-Kyu Han, Yun Suk Huh, Arindam Ghosh, Sonu Gandhi

**Affiliations:** 1 Department of Physics, Indian Institute of Science (IISc), Bangalore, 560012 India; 2 Department of Energy and Materials Engineering, Dongguk University, Seoul, 30 Pildong-ro 1-gil, Seoul, 04620 Republic of Korea; 3 Department of Biological Engineering, Inha University, 100 Inha-ro, Nam-gu, Incheon 22212 Republic of Korea; 4 Center for Nanoscience and Engineering, Indian Institute of Science (IISc), Bangalore, 560012 India; 5 DBT-National Institute of Animal Biotechnology (DBT-NIAB), Hyderabad, 500032 Telangana India

## Abstract

Chlorpyrifos is one of the most widely used pesticides that acts on the nervous system by inhibiting acetylcholinesterase. Prolonged use of chlorpyrifos causes severe neurological, autoimmune, and persistent developmental disorders in humans. Therefore, in this study, a highly sensitive and robust biosensor platform was devised by fabricating graphene field effect transistors (graFET) on Si/SiO_2_ substrate for the detection of chlorpyrifos in real samples. Anti-chlorpyrifos antibodies were immobilized successfully on the graphene surface. Under optimal conditions, graFET sensor showed an excellent response for chlorpyrifos detection in the linear range of 1 fM to 1 µM with a limit of detection up to 1.8 fM in spiked samples. The developed graFET biosensor is highly stable, sensitive, and specific for chlorpyrifos as confirmed by its significant ability to detect changes in electrostatic potential. These findings signify useful efficacy of immunobiosensors for the detection of chlorpyrifos and other organophosphates in fruits and vegetables.

## Introduction

The widespread use of pesticides in agriculture has led to pesticide traces in the air, water and soil. The presence of pesticide traces in food and environment poses serious health issues^1^. Chlorpyrifos (O,O-diethyl-O-3, 5, 6-trichloro-2-pyridylphosphorothioate) is an organophosphate that is used worldwide to control pests on fruits, vegetables, including a variety of crops. It has high toxicity and long retention time that act on the nervous system and affects mainly neurological development, as well as causes cancer and reproductive disorders^2–4^.

Currently, detection of these pesticide residues is based on various analytical methods such as liquid chromatography (LC), high-performance liquid chromatography (HPLC), gas chromatography (GC), mass spectrometry (MS), capillary electrophoresis (CE), and enzyme-based assay^5–8^. However, these analytical techniques suffer from several drawbacks such as tedious sample preparation, sophisticated and expensive instrumentation with a consistent need of a trained operator, and are not on-site applicable. Compared to developed analytical methods, these charge sensitive electrochemical sensors offer a variety of advantages such as high sensitivity, quick response, ease of operation and on-site applicability that are promising alternatives for rapid detection of pesticides^9–12^. Enzyme-based sensors are gaining importance due to their high sensitivity and fast response but are limited to a certain class of pesticides^13,14^. Immunosensors work on the principle of antigen-antibody (Ag-Ab) interaction with high specificity and sensitivity that can be used for the detection of pesticides, narcotic drugs, bacteria, and viruses^15–21^. Field effect transistors (FET) are excellent charge sensitive sensors, which can be used for real-time monitoring to study the target-analyte interaction at the electrode surface, and an input signal can be translated into a readable output electrical signal. Liquid state measurements are more desirable than a dry state in FET-based sensors due to real-time detection of biological molecules at less than 1 V, as high temperature can degrade the native state of proteins^22^. FET-based on graphene as the biosensing platform has been used for the detection of target analyte larger than 30 kDa antigens, antibodies or charged molecules^22^. Graphene, a single layer of carbon atom arranged in honeycomb lattice, with a linear, gapless band-structure, shows a plethora of interesting properties like quantum hall effect, large Young’s modulus, thermal conductivity, etc. as well as offers several advantages for biological applications like high mobility, large surface to volume ratio due to its ability to absorb various aromatic biomolecules by π-π stacking and electrostatic interactions which makes it ideal for biosensor applications^23^. It has also shown immense potential for detecting bioactive molecules such as enzymes, DNA, aptamers, making it important for industrial use as well^24–30^.

Here, we report the detection of chlorpyrifos pesticide on functionalized graphene-FET-based immunosensor. The graphene-FET is fabricated using exfoliation technique on Si/SiO_2_ substrate followed by thermal evaporation of Cr/Au (5/50 nm) electrodes after electron beam lithography and has been used for the detection of chlorpyrifos (Chl) with a limit of detection (LOD) of 1.8 fM in standard samples that is 10-fold (an order of magnitude) less than the previous reports^31^, making them one of the most sensitive detectors of chlorpyrifos till date. Along with ease of fabrication, the developed devices are highly sensitive, cost effective, can detect minute changes in antigen concentrations, and can be easily integrated into electronic chips for real-time sensing for on-site applications.

## Methods

### Labeling and Immobilization of chlorpyrifos antibody on FET device

Chl-Ab was labelled with graphene via carbodiimide chemistry. For this, 0.5 mg of Ab was added dropwise to 75 μM EDC (1-ethyl-3-(3-dimethylaminopropyl) and 75 μM NHS (N-hydroxysuccinimide) solution. The reaction was incubated at RT for 2 h and centrifuged at 10,000 rpm for 15 min at 4 °C and pellet was redissolved in 1 mL of phosphate buffer (PB), pH 7.4. 100 μg of chl-Abs were added dropwise to freshly activated graphene and incubated at RT for 30 min followed by incubation O/N at 4 °C. The graphene (gra) labelled Abs were further characterized to confirm the binding by spectroscopic and microscopic techniques.

### Characterization of chlorpyrifos antibody labelled with graphene

The morphological studies of graphene and gra-chl-Abs nanocomposites were done with scanning electron microscope (SEM) (Hitachi S-3400N). The samples for SEM imaging were made electroactive by coating with gold using Quorum SC7620 sputter coater. Atomic force microscopy (AFM) was performed on Bruker Dimension Icon. Fourier transformed Infra-red (FTIR) spectroscopy was carried out using Shimadzu 8700 FTIR. Optical characteristics were studied by UV-Vis spectrophotometer using (Shimadzu 2600).

### Fabrication of FET device

To fabricate FET devices, we have used mechanically exfoliated graphene, which is known to perform at very low noise levels^32–35^. Exfoliation was performed on Si/SiO_2_ substrates using scotch tape (3 M magic tape) technique where 285 nm SiO_2_ forms back-gate dielectric. Suitable graphene flakes were identified by an optical microscope (Olympus BX51) followed by spin coating and baking of one layer each of PMMA 495 and PMMA 950 at 150 °C that formed the positive resist for e-beam lithography performed in Raith Pioneer to pattern the contact pads. MIBK and IPA (1:3) were used to develop polymer layer post e-beam lithography. Afterwards 5/50 nm of Cr/Au were thermally evaporated to form the source-drain electrodes. The sample was then mounted on ceramic chip carrier using silver epoxy. The contact pads were ball bonded in TPT HB05 wire bonder machine.

### Electrochemical characterization of immobilized chl-Abs on fabricated FET device

All electrical measurements were done using SR 830 Lock-In Amplifier. Keithley 2400 source meter was used to provide back gate voltage. The chl-Abs were immobilized via carbodiimide activation reaction that activates the carboxylic groups on graphene surface and binds via amine groups present on antibodies. The immobilization and hybridization of chl-Abs on FET electrode was studied at different stages by recording changes in resistance. The immobilization of chl-Abs (1 ng/mL) was done in 50 mM PB (pH 7.4). The washing with PB was done at each step to remove excess and unbound material. Chl-Ag was added at different concentrations (1 fM to 1 µM). Real-time sensing in liquid state was done by applying a constant current of 100 nA across the sample and monitoring the resistance change in the FET for various antigen (chl-Ag) concentrations.

## Results and Discussion

### Characterization of chl-Abs immobilized gra-FET immunosensor

**Figure 1:**
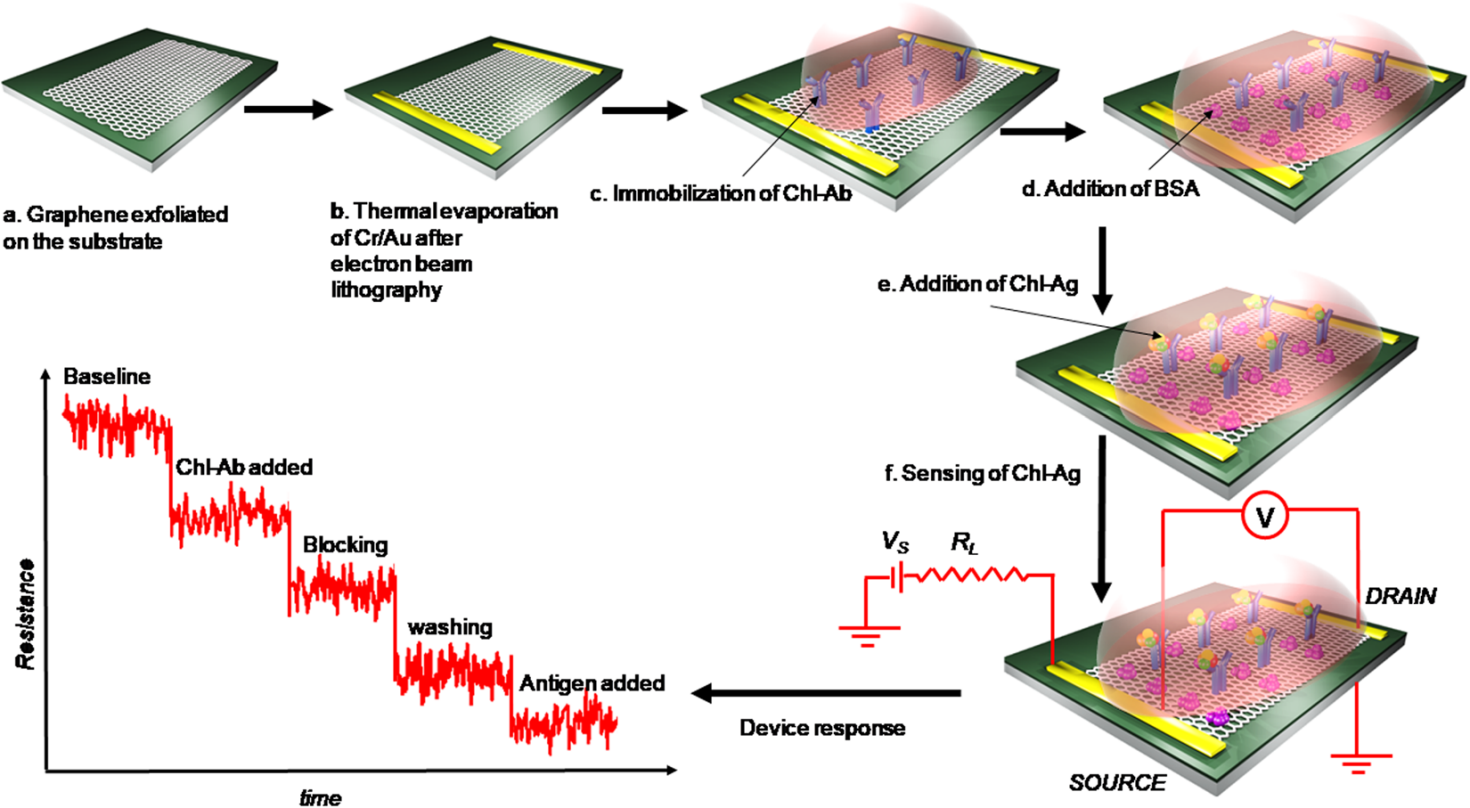
Fabrication procedure for graphene FET device. (**a**) Graphene exfoliated on 285nmSiO_2_/Si substrate using scotch tape technique where SiO_2_ forms the back gate dielectric; (**b**) the graphene was electrically contacted by thermally evaporating 5/50 nm Cr/Au after electron beam lithography; (**c**) chl-Abs were immobilized onto graphene by carbodiimide activation that helps in the binding of the antibodies by covalent bonding; (**d**) Blocking was done with BSA in Phosphate Buffer, pH 7.4; (**e**) biosensing was performed by adding chl-Ag to the micro-device; (**e**) for measuring the response, a constant current circuit was used where V_s_ is the applied source voltage and R_L_ is the current limiting resistance; and (**f**) the signal was measured by monitoring the resistance as a function of time for different concentrations of antigens.

Functionalization of graphene was achieved by carbodiimide chemistry that resulted in the activation of carboxylic groups in graphene and helps in covalent bond formation with amine reactive chl-Abs. The fabrication of functionalized graphene for sensing chl-Ag was done with FET immunosensor. While blocking non-specific sites with BSA on the surface of graphene, chl-Abs was immobilized and specific Ag added on the electrode surface. Change in resistance (%R) was monitored w.r.t. increase in chl-Ag concentration (Fig. 1).

**Figure 2:**
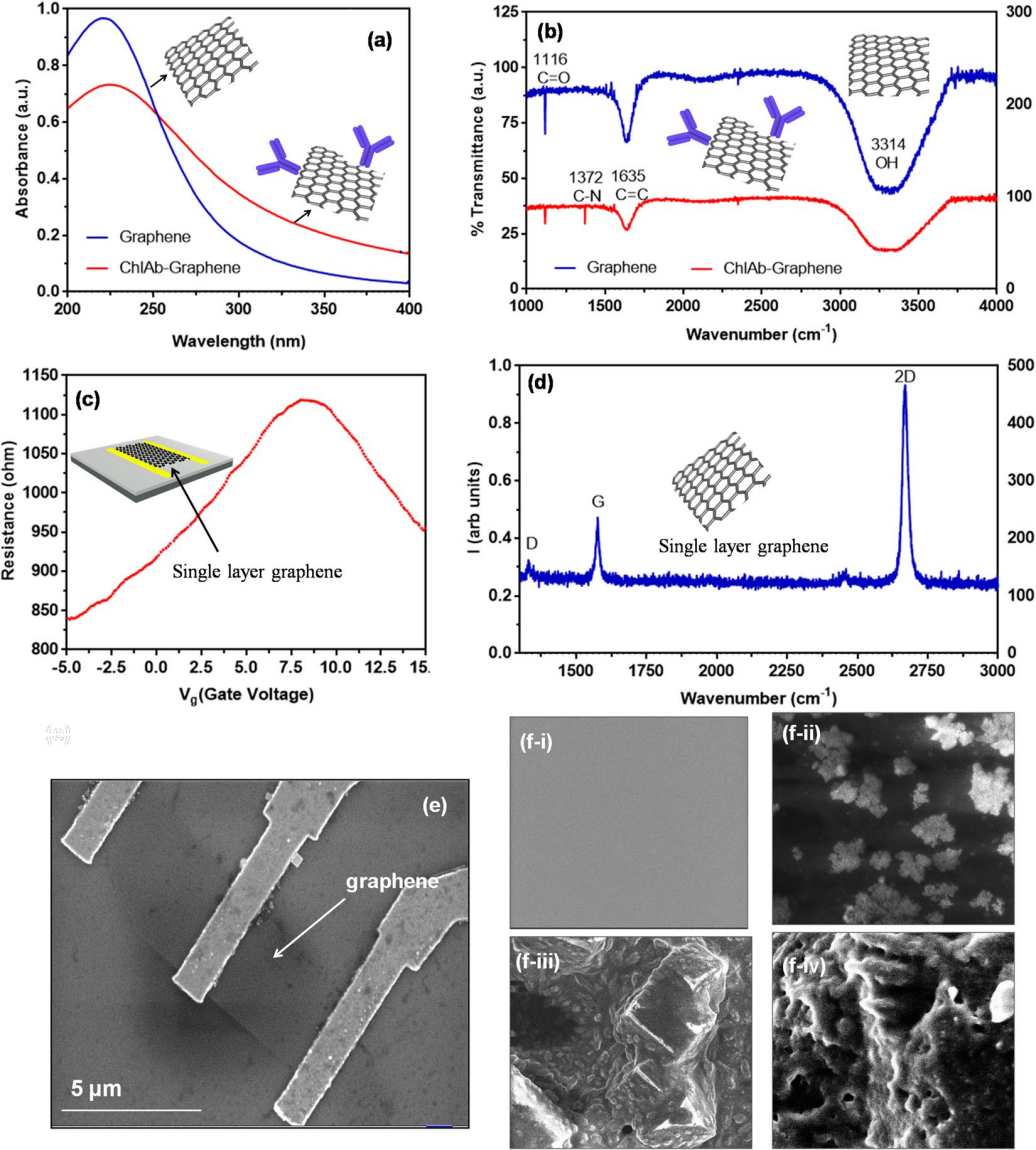
(**a**) UV-Vis spectra of graphene (peak at 230 nm) and graphene-chl-Ab (peak at 240 nm); (**b**) FT-IR spectrum of graphene (red) and graphene labelled with chl-antibody (blue) confirmed the labelling by showing a peak of 1372 cm^−1^ for C-N while 1116 and 1635 cm^−1^ peaks are shared in both cases for C=O and C-C, respectively; (**c**) R vs Vg of the FET showing ambipolar transport; (**d**) Raman spectrum of the graphene used in the FET showing characteristic 2D peak at ~2600 cm^−1^ confirming the single layer nature; (**e**) SEM micrograph of device showing gold electrode connected with the surface of graphene. The red dashed line showed the boundary of graphene. The scale bar is 5 μms; (**f**-**i**) SEM morphology of graphene, (**f-ii**) graphene-Chl-Ab (white globular structures), (**f-iii**) graphene-Chl-Ab blocked with BSA, and (**f-iv**) graphene-Chl-Ab bound with Chl-Antigen (Ag).

To check the suitability of graphene for chlorpyrifos detection, binding of graphene with chl-Abs was first studied (see supplementary information). UV-Vis spectra (Fig. 2a) showed the peak at 230 nm for graphene (blue line) while red shift observed at 235 nm after binding with chl-Abs (red line). FT-IR spectra showed (Fig. 2b) peaks at 1116 and 1635 cm^−1^ for C=O and C-C stretch, respectively. Presence of peak at 1372 cm^−1^ for C-N confirmed that binding occurred between graphene and chl-Abs. The resistance (R) vs gate voltage (Vg), measured in constant current mode, showed the characteristic bell-shaped curve with a maxima at ~8 V that corresponds to charge neutrality point or Dirac point (Fig. 2c). The transfer characteristics before and after antibody interaction clearly shows a shift in the graphene Dirac point^19^. The shift in the Dirac point indicates a change in the number density of the graphene channel, due to antibody-antigen interaction, which has led to charge redistribution of the graphene channel. The Raman spectra (Fig. 2d) displayed the characteristic sharp 2D peak for single layer graphene (SLG) with the ratio of I_2D_/I_G_ ~ 2. The mobility of device obtained from *σ* vs *n* was approximately 750 cm^2^/Vs Scanning electron micrograph (SEM) of SLG showed a clear graphene layer (Fig. 2e). Figure 2f showed morphology of (i) graphene after binding of (ii) chl-Abs with graphene (while globular structures), reconfirmed the immobilization on the graphene electrode. Further passivation of unobound sites with BSA can be seen in Fig. 2f-iii and binding with chlorpyrifos antigen with specific antibody (Fig. 2f-iv).

### Binding characteristics of chl-Abs

**Figure 3:**
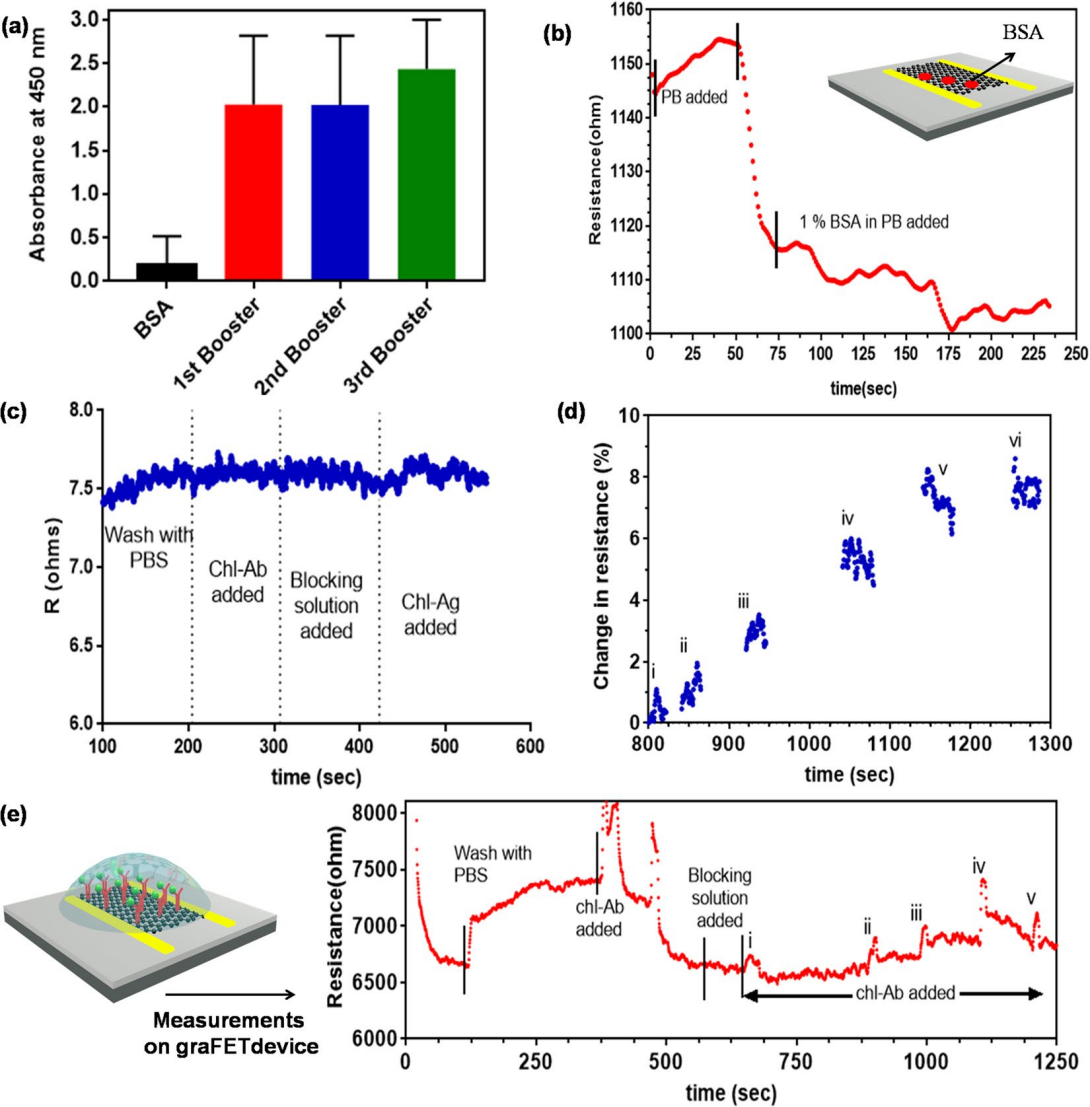
(**a**) Binding of chl-Abs with BSA, and chl-BSA. The concentration of Chl-Abs was 0.1 μg/mL; (**b**) R of graphene channel showed a dip when reacting with 1% BSA in PB. (**c**) The change in resistance was recorded on the gold surface without graphene does not show antibody binding, reconfirmed that signal obtained from graphene network; (**d**) Calibration curve resultant from the immunoreactions showed the % R as a function of time for different concentrations of chl-Ag. (**e**) Schematic representation of Chl-Abs-gra-FET sensor; Kinetic response from the gra-FET at each step and after exposure of free chl at different concentrations: (i) 1 fM (ii) 10 fM (iii) 100 fM (iv) 1 pM (v) 10 pM, and (vi) 100 pM in PB, pH 7.4.

Figure 3a showed the gra-FET immunosensor platform. To develop a specific and sensitive assay for small molecules is an important aspect. The main step is to synthesize appropriate antibody that is specific and sensitive towards the target biomolecules. Therefore, chl-BSA conjugate was used to generate Abs in rabbit. The developed antibodies showed high specificity for chl upto the dilution of 1:640,000. From the binding assay, it was confirmed that the developed Abs are mainly produced against chl not against BSA (Fig. 3a). The phenomenon can be explained as there might be a uniform distribution of haptens conjugated with the carrier protein (BSA) that helped in the recognition of hapten (chl) by the immune system of animal^10,16^.

### Electrochemical performance of fabricated gra-FET electrode system

Graphene was functionalized with EDC/NHS for activation of carboxylic groups for the development of grapheneFET-basedsensor^22^. Blocking unbound surface of graphene was done with BSA and the change in the resistance was monitored (Fig. 3b). To reconfirm the change in resistance obtained from graphene layer but not from gold surface. The biomolecules (Antibody-BSA (for blocking)-chl-Ag) were immobilized on gold surface and the signal was recorded that showed no change in resistance at each step of binding. This process again confirmed that the change in resistance was due to graphene but not from bare gold (Fig. 3c). For the quantitative estimation of detection capabilities of FET device, the graphene channel resistance was monitored continuously at each step to estimate the sensing action of FET for various chl-Ag concentrations. The graphene channel functions as a transducer that converted a chemical signal from the biomolecules and acted as receptor in this case, into a measurable electrical signal that could be read by the lock-in amplifier. The diffusive motion of carriers in the channel is captured by the Drude conductivity expression, which is given by *σ* = *neμ*, where σ, n, and μ are the conductivity, carrier density and mobility of the carriers respectively. The Ag-Ab interaction at the reactive site leads to doping of graphene channel due to heterogeneous electron transfer, which was manifested as a change in graphene channel resistance, since $\sigma \,\propto 1/R$. The sensitivity of the devices were determined by extracting the % change in R, which was obtained by taking the R of the channel with a buffer solution as the baseline. The calibration curve of the immunoassay developed at various chl-Ag concentrations is shown in Fig. 3d. A measurable change in R was recorded for concentrations as low as 1.8 fM. For chl-Ag concentration at 10 pM after that the resistance saturates, probably because all the sites in chl-Abs have been occupied with chl-Ag (CITE). Kinetic studies were performed, and exponential sensogram fit plotted for chl (Fig. 3e). The spikes in the resistance may originate due to changes in the local electrostatic environment, which is a transient state when the system is out of equilibrium. The spiked chlorpyrifos was prepared in linear range of 1 fM to 1 µM with detection range from 1.8 fM to 100 pM. The gra-FET-based sensor for chl detection showed a significant enhancement in the limit of detection (LOD) of 1.8 fM for chl-Ag-Ab complex, compared to other devices (Table 1), making it one of the most sensitive detectors till date. The LOD can be further enhanced, possibly by using large area chemical vapour deposition (CVD) grown graphene. The larger area can lead to an enhancement in the number of active sites on functionalization, and an increased LOD. Since graphene is highly stable and has a low environmental impact, such functionalized graphene FET-based devices can be easily integrated into an electronic chip for large-scale field applications for detection of not just chlorpyrifos but other specific biomolecules as well.

**Table 1:** Comparison of developed electrochemical sensors for chlorpyrifos with its limit of detection.

Nanomaterials	Method	Limit of detection (LOD)	References
ZnS nanoparticle	Amperometry	1.5–40 × 10^−9^ M	^26^
Single walled carbon nanotubes	Voltammetry	1 × 10^−12^ M	^33^
Exfoliated graphite nanoPlatelet (xGnPs)–chitosan cross-linked composite	Voltammetry	1.58 × 10^−10^ M	^34^
ZrO_2_/RGO	Amperometry	10^−13^ M	^25^
Single layer graphene FET	Electrical transport	10^−15^ M	Present study

## Conclusions

In conclusion, we have developed graphene-based FET immunosensor for the detection of widely used chlorpyrifos pesticide. The detection, which was performed by monitoring the change in resistance of the funtionalized-graphene channel, for several antibody resistance, yielded a label-free detection of chlorpyrifos with very low detection limit of 1.8 fM in standard buffer. The results of our measurements clearly indicate the choice of using graphene channel as a detector for real-time sensing of very low concentrations of chlorpyrifos, and should be further exploited for providing a low cost platform for on-field surveillance. The limit of detection can be further enhanced by increasing the active area of interaction by employing a large area, CVD-grown graphene. The established detection limits for chlorpyrifos by World Health Organization (WHO) is 2 g/kg, therefore the developed graphene-based ultrasensitive immunosensor can be used for detection of chlorpyrifos in fruits and vegetables.

## Electronic supplementary material

Supplementary Information
