# Supplementary material for: Microfluidic-based graphene field effect transistor for femtomolar detection of chlorpyrifos
Source: Sci Rep. 2019 Jan 22;9:276. doi: 10.1038/s41598-018-36746-w (PMC6343030; doi:10.1038/s41598-018-36746-w)
Supplement: Supplementary file 1 — Supplementary Information [file 41598_2018_36746_MOESM1_ESM.docx]

**Microfluidic-based graphene field effect transistor for femtomolar detection of chlorpyrifos**

Saurav Islam^1,┼,*^, Shruti Shukla^2^, Vivek K. Bajpai^2^, Young-Kyu Han^2^, Yun Suk Huh^3*^, Arindam Ghosh^1,4^ & Sonu Gandhi^5,┼,*^

^1^Department of Physics, Indian Institute of Science (IISc), Bangalore-560012, India

^2^Department of Energy and Materials Engineering, Dongguk University, Seoul, 30 Pildong-ro 1-gil, Seoul 04620, Republic of Korea

^3^Department of Biological Engineering, Inha University, 100 Inha-ro, Nam-gu, Incheon 22212, Republic of Korea

^4^Center for Nanoscience and Engineering, Indian Institute of Science (IISc), Bangalore-560012, India

^5^DBT-National Institute of Animal Biotechnology (DBT-NIAB), Hyderabad-500032, Telangana, India

***Correspondence:**

**Dr. Sonu Gandhi**; E-mail: [sonugandhi@gmail.com](mailto:sonugandhi@gmail.com)

**Saurav Islam**; E-mail: [isaurav@iisc.ac.in](mailto:isaurav@iisc.ac.in)

**Dr. Yun Suk Huh**; E-mail: [yunsuk.huh@inha.ac.kr](mailto:yunsuk.huh@inha.ac.kr)

^┼^ Sonu Gandhi and Saurav Islam contributed equally to this work.

**Running head:** FET immunosensor for chlorpyrifos detection

**Methods**

**Reagents.**

Single crystals of Kish graphite used for exfoliation purpose were purchased from Covalent Materials Corporation. PMMA used as a resist in lithography, was purchased from MicroChem Corp. Si/SiO_2_ substrate was sourced from Nova Electronic Materials. Chromium and gold for thermal evaporation were purchased from Kurt J. Lesker Company. Scotch tape used for exfoliation was made by 3M.

Chlorpyrifos-Ovalbumin (Chl-OVA) and chlorpyrifos-Bovine serum albumin (Chl-BSA) conjugates were purchased from Creative Diagnostics, USA. Chlorpyrifos, atrazine and 2, 4-D was purchased from Sigma Aldrich. Complete Freund’s adjuvant (CFA), incomplete Freund’s adjuvant (IFA), bovine serum albumin (BSA), sodium dihydrogen phosphate (NaH_2_PO_4_), disodium hydrogen phosphate (Na_2_HPO_4_), 1-ethyl-3-(3-dimethylaminopropyl) carbodiimide hydrochloride (EDC), and N-hydroxysulfosuccinimide (sulfo-NHS) were purchased from Sigma Aldrich.

**Antibody generation and its purification**.

6-8 weeks old New Zealand white rabbit was immunized subcutaneously with 250 mg chl-BSA conjugate. During first booster, the conjugate was mixed with equal volume of complete Freund’s adjuvant (CFA) followed by incomplete Freund’s adjuvant (IFA) in subsequent boosters after 21 days. Blood was collected after fifth day of each booster, serum precipitated, and antibodies were purified by affinity chromatography as per the standard protocol. The concentration of chl-Abs was determined at 280 nm after overnight (O/N) dialysis in 1x PBS (pH 7.4) at 4 ^0^C. The antibodies were stored at -20 ^0^C for long term storage. All experiments were done after approval from animal ethics committee of CPCSEA (Committee for the Purpose of Control and Supervision of Experiments on Animals) of the institute. All methods were performed in accordance with the relevant guidelines and regulations.

**Immunoassay development***.*

96 well ELISA plates were coated with (100 μL/well) chl-OVA conjugate at 5 μg/mL. The plates were incubated O/N at 4 ^0^C. The blocking was done with 5% skim milk in 1X PBS, pH 7.4 and incubated for 30 min at 37 ^0^C. Chl-Abs (100 μL/well) were added at serial dilution (upto 1:640,000) to confirm the binding with chl and incubated for 1h at 37 ^0^C. Goat anti-rabbit IgG-HRP was added (100 μL/well) at fixed 1:10,000 dilution and incubated for 1h at 37 ^0^C. TMB substrate (100 μL/well) was added and incubation was done for 15 min at room temperature (RT) followed by addition of 1N H_2_SO_4_ (50 μL/well) to stop the reaction. The absorbance was taken at 450 nm using Thermo Fisher ELISA Reader.
